# Supplementary material for: Aerosol-Mediated Spread of Antibiotic Resistance Genes: Biomonitoring Indoor and Outdoor Environments
Source: Int J Environ Res Public Health. 2024 Jul 27;21(8):983. doi: 10.3390/ijerph21080983 (PMC11353316; doi:10.3390/ijerph21080983)
Supplement: Supplementary file 1 [file ijerph-21-00983-s001.zip › ijerph-3107139-supplementary.pdf]

Table S1. Literature review ARGs in aerosols.

Site	Location	Sample type	Site description	Method	Sampler	Size Fraction	Reference
Public Building, Hospital	Indoor and Outdoor	Aerosols	KISR, Kuwait, urban hospitals	HT-qPCR (Qiagen)	Customized sampler	< 0.22 µM	[58]
Hospital	Indoor and Outdoor	Dust and air particulate matter	Urban hospital	HT-qPCR (Takara) and 16S rRNA targetted amplicon sequencing	portable atmospheric particulate matter samplers	$PM_{2.5}$, and PM_{10},	[51]
Public Building	Indoor and Outdoor	Aerosol	Dormitories, offices Nankai University	Microbiology and 16S rRNA targetted amplicon sequencing	High-volume tandem liquid impinging sampler		[69]
Public Building, Hospital, Animal Hospital	Indoor	Swabs	Medical centre, veterinary hospital, office building, Ohio State University, Columbia	qPCR (selected genes), 16S rRNA targetted amplicon sequencing			[60]
Hospital	Indoor/outdoor interface	Aerosols	Outpatient department (3.72 million outpatients), urban centre surrounded by malls and too many vehicles (35,000 people per km^2), suburban centre with very less vehicles (2800 people per km^2)	Shot-gun metagenomics	Automatic Flow Control Medium Volume TSP Sampler	$PM_{2.5}$, and PM_{10}	[57]
Public Buildings	Indoor	Bioaerosols	Office rooms, Southern Poland	Microbiology/AST and multiplex qPCR	Anderson six-stage cascade impactor	7.0, 4.7, 3.3, 2.1, 1.1 and 0.65 µm	[67]
Public Building	Indoor	Bioaerosols	Office rooms, Warsaw, Poland	Microbiology/AST			[126]
Public Building	Indoor	Bioaerosols	High school gym	Microbiology/AST	Anderson six stage cascade		[68]
Waste management sites	Ambient	Bioaerosols	Waste sorting plant, south Poland	Microbiology/AST	Anderson six-stage cascade	7.0, 4.7, 3.3, 2.1, 1.1 and 0.65 µm	[127, 128]

Waste management sites	Ambient	Aerosols	Wastewater Treatment Plant, Hong Kong	qPCR and shot-gun Metagenomics	High Volume air sampler	$PM_{2.5}$	[75]
Polluted river sites	Indoor and Outdoor	Aerosols	The capital city of Beijing in northern China, the Yangtze River Delta (YRD) in eastern China, and the Pearl River Delta (PRD) in southern China	RT-PCR, 16S 16S rRNA targetted amplicon sequencing		$PM_{2.5}$	[82]
Agricultural and	Ambient	Bioaerosols	Agricultural sites, California	AST and HT-qPCR			[79]
Waste management sites	Ambient	Bioaerosols	Waste recycling sites	Digital droplet PCR			[71]
Public buildings	Ambient	Bioaerosols	Harbin	qPCR (selected genes)	-	$PM_{2.5}$	[129]
Waste management sites		Aerosols	Commercial areas, educational areas, residential areas and WWTP, Shijiazhuang, Hebei, China	AST and HT-qPCR		$PM_{2.5}$, and PM_{10}	[43]
Polluted river waters		Bioaerosols	Impacted urban surface waters, La Paz Bolivia	ddPCR			[81]
Animal Farms	Outdoor	Aerosols	Swine manure biogas digestate	qPCR			[76]
Animal Farms	Indoor and outdoor	Aerosols	Chicken farm, Henan	HT-qPCR	Anderson six stage		[86]
Public buildings	Ambient	Bioaerosols	Fine hazy and dusty weather urban city of Tianjin, China	HT-qPCR (Wafergen Smart Chip Real-time PCR system), selected carbapenem and ampicillin-resistant bacteria through microbiology technique, 16S rRNA targetted amplicon sequencing	High volume tandem liquid impinging sampler	0.22 µM	[130]

Waste management sites	Ambient	Aerosols	Ambient air above a WWTP	Metagenomics		$PM_{1.0}$	[41]
Waste management sites	Ambient	Bioaerosols	Open Waste Canals, Kanpur, India	Microbiology and ddPCR	Dry Filter Air Sampler		[37]
Public building	Polluted air	Bioaerosols	Beijing	AST and HT-qPCR	Portable high-volume air sampler		[83]
Animal farm	Indoor and outdoor	Bioaerosols	Poultry farm	RT-PCR, 16S amplicon sequencing	transportable high-flow bioaerosol sampler		[132]
Waste management sites	Indoor and outdoor	Bioaerosols	up- and downwind, composting, packaging, and office areas of four composting plants, Beijing China	ddPCR, 16S amplicon sequencing	total suspended particulate (TSP) impactors		[63]
Animal farms, public buildings, forests	Indoor and outdoor	Bioaerosols	Indoor (swine, dairy, clinics, homeless shelter) outdoor (urban, semi-urban, livestock, alpine forest) Colorado	qPCR (selected genes)	Liquid capture method, virtual impaction method,	PM_{10} (5-10 µm particles)	[49]
Animal farms	Indoor and outdoor	Bioaerosols	Chicken and fish waste	qPCR (selected genes)	FA-1 six-stage impact microbial sampler	0.65–1.1 µm, 1.1–2.1 µm, 2.1–3.3 µm, 3.3–4.7 µm, 4.7–7.0 µm, >7.0 µm	[133]
Remote mountains	Outdoor	Atmospheric deposition (rain/snow)	Remote mountains of Pyrenes, Spain	qPCR (selected genes), 16S amplicon sequencing	Wet atmospheric collector		[84]
Agricultural sites	Indoor	Aerosol	Greenhouse	RT-PCR		$PM_{2.5}$, and PM_{10}	[134]
Public buildings	Ambient	Aerosols	Urban sites, Xiamen, China	HT-qPCR	High-volume air sampler	$PM_{2.5}$, and PM_{10}	[80]

Residential buildings	Outdoor	Dust	Windowsills of buildings with plants growing	HT-qPCR			[135]
Hospitals	Indoor	Swabs	Hospital	Culture-based metagenomics			[54]
Public buildings		Air	Urban community farmers market	qPCR (selected genes)	High-volume air sampler	$PM_{2.5}$	[136]
			Ocean-atmosphere	qPCR			[137]
Waste management sites	Outdoor	Aerosols	Wastewater treatment plants	qPCR (selected genes)	laboratory-scale spray tank chamber		[77]
Animal Farms	Ambient	Aerosols and dust	Chicken and dairy farms	qPCR (selected genes)	portable high-volume sampler collected on nutrient agar for aerosols; dust was collected through sterile brushes		[64]
Waste management sites	Indoor and outdoor (vent)	Bioaerosols	Municipal solid waste transfer station	Metagenomics	Medium flow samplers	≤10 µm	[74]
Animal farms		Bioaerosols	Pig farms	qPCR (selected genes)	Medium volume samplers	$PM_{2.5}$	[138]
Animal farms		Bioaerosols	Hen layer house	Metagenomics			[139]
Waste management sites		Bioaerosols	Aeration tanks of municipal sewage treatment plant	qPCR assay (Qiagen), culture-dependent and culture-independent	Air scan sampler		[78]
Animal farm		Air particulate matter	Swine, cattle, layer and broiler farms	ddPCR	Medium volume samplers, Anderson six-stage sampler	0.65–1.1 µm, 1.1–2.1 µm, 2.1–3.3 µm, 3.3–4.7 µm, 4.7–7.0 µm, >7.0 µm	[140]
Animal farm			Farm air				[141]

Animal farm			Surrounding air of livestock (pig) farms				[12]
Animal farm<br>Public building (Res Labs)	Indoor and outdoor	Air	Animal biology laboratories	AST and qPCR	High volume air samplers		[70]
		Air particulate matter	Smog events	Metanalysis of metagenomic sequences		$PM_{2.5}$, and PM_{10}	[131]
Hospital		Swabs	Tertiary Care Hospital, Singapore	Metagenomics			[59]
Hospitals	Indoor	Bioaerosols	transfusion area, out-patient department, and inpatient department<br>Urban hospitals of China	Microbiology, qPCR (selected genes)	high-volume total sus- pended particle (TSP) sampler		[53]
Public parks	Outdoor	Air	Public parks, California		Pumps connected to polycarbonate filters		[11]
Hospitals, animal farms, public buildings	Indoor	Dust	Air conditioning systems of hospitals, farms and residences	HT-qPCR			[55]
			Urban Air, Beijing	Metanalysis of Metagenomic sequences			[142]
Animal hospital	Indoor	Surface swabs	Veterinary teaching hospital	Oxford nanopore sequencing, Illumina sequencing, targeted qPCR			[62]
Hospital		Air dust	Outpatient hall, ophthalmology, pediatrics and inpatients departments of a hospital	qPCR and metagenomics	Air conditioner strainers		[56]
Hospital		Surface swabs	Patients room, floor, doorhandle and sink, neurology ward at the Charité–Universitätsmedizin Berlin	RT-PCR			[61]
Hospital	Ambient and indoor	Bioaerosols	Urban Hospital	Metagenomics	High volume samplers	$PM_{2.5}$	[72]

Public buildings, hospitals, public parks		Dust	Densely populated urban places (malls, hospitals, schools, parks)	HT-qPCR, 16S amplicon sequencing		[52]
Hospital	Indoor	Bioaerosols	Pulmonology unit,	Endpoint PCR	Swabs, Anderson 6 stage impactors with media, gelatin membranes	[50]
Animal farms and Waste management sites, Urban	Ambient	Total suspended particles	a pig farm, a chicken farm, a WWTP and the downtown area in Zhuhai, South China	Shot gun metagenomics	High volume samplers	[73]

HT-qPCR-high throughput quantitative polymerase Chain reaction; PCR-polymerase chain reaction, RT-PCR- reverse transcription polymerase chain reaction; qPCR-quantitative polymerase chain reaction; AST-antibiotic susceptibility assay.

Table S2: Studies included for metanalysis

Location	Sub-location	Total number of ARGs	Relative Abundance (copies/16S rRNA)	Reference
Zhuhai, South China	chicken farm	255	0.66	[73]
	pig farm	230	0.85	
	WWTP	183	0.46	
	downtown		0.07	
Shenzhen hospital Peking University	pediatrics	167	0.00064	[56]
	outpatients	42	0.00032	
	outpatient's hall	144	0.00028	
Hen farms in Hebei		192	1.25	[138]
Municipal solid waste transfer station		265	2.09	[74]
WWTP Jinan, China		40		[41]
Urban	hospital	11	-0.72	[72]
	urban		0.03	
WWTP HongKong	WWTP	75	-0.18	[75]
	urban	36	-0.16	
	coastal	5	-0.15	
City hospital, China	hospital	145	0.11	[57]
	urban	29	0.11	
	sub-urban	37	0.008	
